# A Joint Evaluation of Neurohormone Vasopressin-Neurophysin II-Copeptin and Aortic Arch Calcification on Mortality Risks in Hemodialysis Patients

**DOI:** 10.3389/fmed.2020.00102

**Published:** 2020-03-31

**Authors:** Jia-Feng Chang, Yu-Shao Chou, Chang-Chin Wu, Po-Cheng Chen, Wen-Chin Ko, Jian-Chiun Liou, Chih-Yu Hsieh, Wei-Ning Lin, Li-Li Wen, Shu-Wei Chang, Tao-Hsin Tung, Ting-Ming Wang

**Affiliations:** ^1^Division of Nephrology, Department of Internal Medicine, Shuang Ho Hospital, Taipei Medical University, New Taipei City, Taiwan; ^2^Graduate Institute of Aerospace and Undersea Medicine, Academy of Medicine, National Defense Medical Center, Taipei, Taiwan; ^3^Department of Nursing, Yuanpei University of Medical Technology, Hsinchu, Taiwan; ^4^Division of Nephrology, Department of Internal Medicine, En Chu Kong Hospital, New Taipei City, Taiwan; ^5^Renal Care Joint Foundation, New Taipei City, Taiwan; ^6^College of Medicine, Fu Jen Catholic University, New Taipei City, Taiwan; ^7^Department of Emergency Medicine, En Chu Kong Hospital, New Taipei City, Taiwan; ^8^Department of Orthopaedic Surgery, School of Medicine, National Taiwan University, Taipei, Taiwan; ^9^Department of Orthopaedic Surgery, National Taiwan University Hospital, Taipei, Taiwan; ^10^Department of Orthopedics, En Chu Kong Hospital, New Taipei City, Taiwan; ^11^Department of Biomedical Engineering, Yuanpei University of Medical Technology, Hsinchu, Taiwan; ^12^Department of Urology, En Chu Kong Hospital, New Taipei City, Taiwan; ^13^Division of Cardiology, Department of Internal Medicine, Cathay General Hospital, Taipei, Taiwan; ^14^School of Biomedical Engineering, Taipei Medical University, Taipei, Taiwan; ^15^Department of Pathology, National Defense Medical Center, Tri-Service General Hospital, Taipei, Taiwan; ^16^Graduate Institution of Biomedical and Pharmaceutical Science, College of Medicine, Fu Jen Catholic University, New Taipei City, Taiwan; ^17^Department of Medical Laboratory Science and Biotechnology, Yuanpei University, Hsinchu, Taiwan; ^18^Department of Clinical Laboratory, En Chu Kong Hospital, New Taipei City, Taiwan; ^19^Department of Civil Engineering, National Taiwan University, Taipei, Taiwan; ^20^Department of Medical Research and Education, Cheng Hsin General Hospital, Taipei, Taiwan

**Keywords:** vasopressin, neurophysin II, copeptin, vascular calcification, mortality, dialysis

## Abstract

**Objective:** Systemic hypoperfusion is intricately involved in neurohormone secretion, vascular calcification (VC) related impaired vasodilation, and luminal stenosis. We aimed to conduct a joint evaluation of vasopressin-neurophysin II-copeptin peptide (VP) and advanced aortic arch calcification (AAC) on all-cause and cardiovascular (CV) mortality in maintenance hemodialysis (MHD) patients.

**Methods:** Unadjusted and adjusted hazard ratios (aHRs) of mortality risks were analyzed for different groups of VP and AAC in 167 MHD patients. The modification effect between higher VP and advanced AAC on mortality risk was examined using an interaction product term.

**Results:** Interactions between VP and AAC with respect to all-cause and CV mortality were statistically significant. In multivariable analysis, higher VP predicted all-cause and CV mortality [aHR: 2.2 (95% confidence interval (CI): 1.1–4.5)] and 2.6 (95% CI: 1.1–4.6), respectively. Advanced AAC was associated with incremental risks of all-cause and CV mortality [aHR: 2.1 (95% CI: 1.1–4.0)and 2.5 (95% CI: 1.0–4.3), respectively]. Patients with combined higher VP (>101.5 ng/mL) and advanced AAC were at the greatest risk of all-cause and CV mortality [aHR: 4.7 (95% CI: 1.2–16.2)and 4.9 (95% CI: 1.1–18.9), respectively].

**Conclusion:** Combined VP and advanced AAC predict not only all-cause but also CV death in MHD patients, and a joint evaluation is more comprehensive than single marker. In light of hypoperfusion and ischemic events in vital organs, VP and AAC could act as more robust dual marker for prognostic assessment.

## Introduction

Patients undergoing maintenance hemodialysis (MHD) are at particular risk for early mortality, and cardiovascular (CV) deaths top the list as the leading cause (1). In light of this, searching for early warning signs with prompt intervention strategies to improve outcomes in MHD population is of prime importance. The hypothalamic-pituitary-adrenal (HPA) axis is stimulated in response to hemodynamic instability and systemic hypoperfusion in the emergency department and intensive care unit (2). Thus, vasopressin related neurohormones have emerged to assist clinicians with diagnosis and risk stratification in myriads of diseases, such as acute kidney injury, acute myocardial infarction (MI), heart failure, lower respiratory tract infection, stroke, and progression of chronic kidney disease (3–8). Furthermore, higher plasma concentrations of copeptin serve as novel biomarkers of combined CV events in MHD patients and significantly correlate with plasma concentrations of arginine vasopressin independent of critical illness (9, 10).

Physiologically, the primary determinant of the vasopressin system is a state of systemic hypoperfusion regardless of cardiac morphological determinants, including myocardial stretch, wall thickness or cavity dimensions (11–14). Nonetheless, MHD patients are especially prone to extraosseous vascular calcification (EOVC) associated luminal narrowing (15), leading to systemic hypoperfusion, reduced vascular compliance, impaired myocardial perfusion, elevated cardiac afterload, left ventricular hypertrophy, and ultimately sudden cardiac death (16–18). With regard to the extremely high prevalence of EOVC in MHD population, emerging evidence have highlighted that surrogate markers of EOVC can independently predict mortality risks (19, 20). Although EOVC can be immediately assessed by diverse diagnostic imaging tools, such as ultrasonography, computed tomography and angiography, chest radiograph has the advantages of being simple, inexpensive, and commonly applicable in daily clinical practice. Advanced aortic arch calcification (AAC) detectable on chest radiograph is identified as a robust predictor of CV mortality beyond traditional risk factors of atherosclerosis (21–24). Despite previous documented implications, the joint and interaction effects of higher plasma concentrations of vasopressin-neurophysin II-copeptin peptide (VP) and advanced AAC on mortality risks remained unclear. Thus, we aimed to conduct a joint evaluation of VP and AAC on all-cause and CV death risks in this population-based study of MHD patients.

## Methods

### Participants in the Cohort

The study had been approved by the Research Ethics Review Committee of En Chu Kong Hospital (ECKIRB1070102) in accordance with the ethical standards of the committee and the Helsinki declaration for research in humans. The written informed consent was obtained from the participants of this study. The relevant details of research methods were described previously (1, 25). Patients undergoing MHD treatment for at least 3 months were eligible for inclusion. All patients had to be older than 18 years of age and be receiving thrice-weekly MHD, and 185 patients were included. Eighteen patients were excluded from the study because of inadequate dialysis, terminal illness, active infections, advanced cancer, active hepatitis, severe protein-energy wasting, incomplete data, or unwilling to participate. Previous CV diseases (CVD) were defined as diseases attributable to myocardial ischemia and infarction, heart failure, symptomatic or life-threatening arrhythmia, cerebrovascular diseases, pulmonary embolism, and peripheral artery diseases.

### Assessment of Exposures

The plasma VP and AAC were assessed at baseline. Plasma concentrations of VP were measured by a commercial quantitative enzyme-linked immunosorbent (ELISA) assay [Human vasopressin-neurophysin 2-copeptin ELISA Kit (Catalog Number: OKEH00396), Aviva Systems Biology, San Diego, CA, USA] in accordance with the manufactures' instructions. The standard chest radiograph was taken in posterior-anterior view. A clinician specializing in thoracic radiology and blinded to patient's clinical data independently reviewed one pre-selected standard chest radiograph obtained from each hemodialysis patient within or as close to the prespecified study period as possible. To determine the severity of AAC detectable on chest radiograph, we used a simple classification system: grade 0 (no visible calcification), grade 1 (small spots of calcification or single thin calcification of the aortic knob), grade 2 (one or more areas of thick calcification, but ≤ 50% of the circular area of the aortic knob), and grade 3 (circular calcification with > 50% of circular area of the aortic knob) (21). All patients were then stratified by AAC grading into the following two groups for statistical analysis: non-advanced AAC, patients with AAC grade 0 or grade 1; advanced AoAC, patients with AAC grade 2 or grade 3.

### Assessment of Covariates

The following bio-demographic and laboratory parameters of each patient were recorded at baseline: age, gender, hypertension, diabetes mellitus (DM), previous CVD, AAC grading, hemodialysis (HD) vintage, systolic blood pressure, diastolic blood pressure, pre-dialysis blood urea nitrogen, normalized protein catabolic rate, creatinine, potassium, calcium, phosphorus, alkaline phosphatase (ALP), alanine aminotransferase, albumin, uric acid, total cholesterol, triglyceride, hemoglobin, platelet, intact parathyroid hormone (iPTH), and VP. HD vintage was defined as the duration of time between the first day of HD treatment and the first day that the patient entered the cohort. Blood pressure was recorded in the horizontal recumbent position before a midweek dialysis session. Pre-dialysis blood samples were obtained from the existing vascular access for further analysis. We adjusted plasma calcium according to the following equation: adjusted calcium, measured calcium+ [(4.0—plasma albumin in g/dL) × 0.8]. All laboratory tests were performed by standard procedures with certified methods.

### Ascertainment of Outcomes

CV mortality in study patients was defined as death attributable to myocardial ischemia and infarction, heart failure, fatal arrhythmia, cardiac arrest because of other causes, cerebrovascular diseases, pulmonary embolism, peripheral artery diseases, and sudden otherwise unexplained death. Non-CV mortality was defined as all other causes of death, i.e., infection, malignancies, gastrointestinal hemorrhage, accidents and miscellaneous. All-cause mortality included CV and non-CV death. Patients were censored at last follow-up, switched to another dialysis unit or received a kidney transplant.

### Statistical Analysis

Continuous variables were presented as mean ± standard deviation, and categorical variables were expressed as number (%). The correlation coefficients between covariates of interest were calculated. The univariate Cox regression analysis was performed to investigate the independence of risk factors associated with all-cause and CV mortality. The included subjects for final analysis were further stratified into higher and lower concentration groups by median values of VP and AAC, respectively. If hazard ratios (HRs) for the variables in the univariate analysis were significant, we would select the covariates into the multivariable regression model. Unadjusted and multivariable adjusted hazard ratios (aHRs) of mortality risks were calculated for different categories of plasma VP and AAC in the Cox regression model, including VP, AAC, DM, CVD, age, albumin, and ALP. The modification effect between plasma VP and AAC on mortality risks was determined using an interaction product term. According to methods previously described, an interaction occurs when the impact of a risk factor on outcome is changed by the value of a third variable, sometimes referred to as effect modification (19, 26).

We evaluated if the effect of VP on mortality risks was modified by AAC through incorporating an interaction term in the multivariable model. The cumulative survival probability and proportional hazards were presented by graphical methods. A significant product term indicates that there is an interaction between VP and AAC on the probability of mortality risk. A *p* < 0.05 was considered statistically significant. We used PASW Statistics SPSS version 22.0 (IBM, NY, USA) to analyze all bio-clinical data of MHD patients.

## Results

The final study sample included 167 MHD patients with complete medical records and follow-up. Baseline bio-clinical data of the whole study population with comparison between survivors and non-survivors are summarized in Table 1. The mean age was 63.3 ± 9.8 years; ~47% were male. Prevalence of DM, hypertension, CVD and advanced AAC were 43.7, 53.3, 47.9, and 48.5%, respectively. The mean duration of follow-up was 26.1 ± 5.9 months. The overall mortality rate was 24.0% during follow-up, corresponding to an annual mortality rate of 11.0%. The incidence of event rate is 110.1 cases per 1,000 person-years. Twenty-eight patients (70.0%) died from CV causes, and 12 (30.0%) non-CV deaths occurred. The bio-clinical parameters that differed significantly between survivors and non-survivors included age, advanced AAC, history of CVD, DM, plasma levels of VP, albumin, ALP, and iPTH. The baseline data of four combined VP and AAC groups were compared and provided in the Table 2. Age, advanced AAC, history of CVD, DM, plasma levels of VP, albumin, and ALP still significantly differed among four combined VP and AAC groups. The comparisons between CV and non-CV deaths are summarized in Table 3. Patients who died as a result of CV death were more frequent to have a history of hypertension, prior CVD, higher levels of VP, albumin and creatinine.

**Table 1:** Bio-clinical data of the whole study population with comparison between survivors and non-survivors.

**Variables**	**Overall (*n* = 167)**	**Survivors (*n* = 127)**	**Deceased (*n* = 40)**
**Age (years)**	**63.3** **±** **9.8**	**61.3** **±** **9.5**	**69.4** **±** **8.4**
Male, *n* (%)	78 (46.7)	64 (50.3)	16 (40.0)
**Diabetes mellitus**, ***n*** **(%)**	**73 (43.7)**	**50 (39.3)**	**24 (60.0)**
Hypertension, *n* (%)	89 (53.3)	65 (51.2)	24 (60.0)
**Advanced AAC**	**70 (41.9)**	**44 (34.6)**	**26 (65.0)**
**Cardiovascular diseases**, ***n*** **(%)**	**80 (47.9)**	**51 (40.2)**	**25 (62.5)**
Systolic blood pressure (mmHg)	137.7 ± 22.6	136.3 ± 21.7	142.2 ± 24.9
Diastolic blood pressure (mmHg)	77.3 ± 11.8	78.4 ± 11.0	74.0 ± 13.6
Hemodialysis vintage (months)	72.3 ± 49.5	68.7 ± 54.3	83.7 ± 27.3
Normalized protein catabolic rate (g/kg/day)	1.0 ± 0.3	1.2 ± 0.3	0.9 ± 0.3
**Albumin (g/dL)**	**3.9** **±** **0.4**	**3.9** **±** **0.4**	**3.6** **±** **0.5**
Total cholesterol (mg/dL)	189.5 ± 48.2	191.1 ± 51.8	182.5 ± 34.1
Triglyceride (mg/dL)	205.2 ± 179.9	214.1 ± 193.7	177.0 ± 124.2
Blood urea nitrogen (mg/dL)	59.3 ± 18.3	58.2 ± 19.1	63.0 ± 15.2
Creatinine (mg/dL)	9.8 ± 1.9	9.8 ± 2.0	10.0 ± 1.6
Uric acid (mg/dL)	7.3 ± 1.2	7.3 ± 1.3	7.4 ± 1.1
Potassium (mmol L^−1^)	4.4 ± 0.8	4.5 ± 0.9	4.3 ± 0.8
Alanine aminotransferase (IU/L)	14.9 ± 12.0	14.3 ± 10.7	17.0 ± 15.4
**Alkaline phosphatase (IU/L)**	**90.1** **±** **24.7**	**87.2** **±** **22.7**	**99.6** **±** **28.5**
Adjusted calcium (mg/dL)	9.3 ± 0.8	9.3 ± 0.8	9.1 ± 0.7
Phosphate (mg/dL)	4.6 ± 1.5	4.6 ± 1.6	4.8 ± 1.5
Calcium-phosphate product	42.9 ± 14.3	42.7 ± 14.6	43.4 ± 13.5
**Intact parathyroid hormone (pg/mL)**	**163.3** **±** **188.6**	**137.3** **±** **133.1**	**245.7** **±** **291.6**
Hemoglobin (g/dL)	10.6 ± 1.2	10.6 ± 1.2	10.5 ± 1.4
Platelet (k/μL)	197.6 ± 63.8	195.5 ± 62.3	204.6 ± 69.0
**VP (ng/mL)**	**116.6** **±** **64.0**	**99.9** **±** **56.0**	**169.8** **±** **57.9**

*Continuous variables were expressed as mean ± SD. Categorical variables are expressed as n (%). Boldface indicates where the values differ significantly between survivors and non-survivors. AAC, aortic arch calcification; VP, vasopressin-neurophysin II-copeptin peptide*.

**Table 2:** Comparisons of baseline bio-clinical data among four combined VP and AAC groups.

**Variables**	**Non-advanced AAC and lower VP**	**Non-advanced AAC and higher VP**	**Advanced AAC and lower VP**	**Advanced AAC and higher VP**
**Age (years)**	**55.9** **±** **6.4**	**57.1** **±** **5.4**	**68.7** **±** **7.7**	**72.7** **±** **6.4**
Male, *n* (%)	17 (38.6)	28 (59.6)	13 (52.0)	20 (39.2)
**Diabetes mellitus**, ***n*** **(%)**	**16 (36.7)**	**11 (23.4)**	**15 (60.0)**	**31 (61.0)**
Hypertension, *n* (%)	24 (54.5)	25 (53.2)	15 (60.0)	25 (49.0)
**Cardiovascular diseases**, ***n*** **(%)**	**12 (27.3)**	**25 (53.2)**	**14 (56.0)**	**29 (56.9)**
Systolic blood pressure (mmHg)	133.7 ± 22.1	138.3 ± 20.8	143.5 ± 24.6	137.7 ± 22.6
Diastolic blood pressure (mmHg)	77.4 ± 9.9	79.8 ± 9.6	73.7 ± 10.4	74.9 ± 15.1
Hemodialysis vintage (months)	69.0 ± 62.3	66.6 ± 45.7	66.8 ± 41.5	83.0 ± 43.3
Normalized protein catabolic rate (g/kg/day)	1.1 ± 0.3	1.1 ± 0.3	1.0 ± 0.2	1.1 ± 0.3
**Albumin (g/dL)**	**4.0** **±** **0.4**	**4.0** **±** **0.3**	**3.8** **±** **0.4**	**3.7** **±** **0.5**
Total cholesterol (mg/dL)	192.6 ± 52.8	188.7 ± 47.1	194.1 ± 58.6	185.2 ± 39.8
Triglyceride (mg/dL)	189.9 ± 142.7	226.3 ± 202.0	274.5 ± 272.1	164.9 ± 110.0
Blood urea nitrogen (mg/dL)	57.3 ± 17.2	59.3 ± 17.7	58.5 ± 18.0	61.8 ± 20.0
Creatinine (mg/dL)	9.4 ± 2.0	10.4 ± 2.2	10.1 ± 1.3	9.6 ± 1.5
Uric acid (mg/dL)	7.3 ± 1.4	7.5 ± 1.3	7.2 ± 0.8	7.1 ± 1.2
**Potassium (mmol L**^**−1**^**)**	**4.6** **±** **0.9**	**4.7** **±** **0.9**	**4.1** **±** **1.0**	**4.3** **±** **0.6**
Alanine aminotransferase (IU/L)	17.7 ± 14.7	13.7 ± 12.3	13.4 ± 12.9	14.4 ± 7.8
**Alkaline phosphatase (IU/L)**	**66.3** **±** **6.3**	**101.6** **±** **16.4**	**71.4** **±** **9.8**	**109.3** **±** **22.9**
**Adjusted calcium (mg/dL)**	**9.4** **±** **0.8**	**9.4** **±** **0.7**	**9.4** **±** **0.9**	**9.0** **±** **0.7**
Phosphate (mg/dL)	4.7 ± 2.1	4.4 ± 1.2	4.5 ± 1.2	4.8 ± 1.4
Calcium-phosphate product	43.8 ± 19.0	41.7 ± 12.7	42.6 ± 12.2	43.2 ± 12.2
Intact parathyroid hormone (pg/mL)	93.2 ± 66.5	192.5 ± 189.1	234.4 ± 325.4	162.0 ± 151.5
Hemoglobin (g/dL)	10.7 ± 0.9	10.8 ± 1.4	10.1 ± 1.1	10.6 ± 1.4
Platelet (k/μL)	199.0 ± 54.8	197.7 ± 72.9	173.4 ± 59.0	205.5 ± 63.3
**VP (ng/mL)**	**75.6** **±** **40.6**	**80.1** **±** **31.1**	**147.1** **±** **63.2**	**170.8** **±** **56.5**

*Continuous variables were expressed as mean ± SD. Categorical variables are expressed as n (%). Boldface indicates where the values differ significantly between survivors and non-survivors. AAC, aortic arch calcification; VP, vasopressin-neurophysin II-copeptin peptide*.

**Table 3:** Comparison of demographic characteristics and relevant laboratory data between CV deaths and non-CV deaths.

**Variables**	**CV deaths (n=28)**	**Non-CV deaths (n=12)**
Age (years)	70.2 ± 9.4	67.4 ± 5.5
Male, *n* (%)	13 (46.4)	3 (25.0)
Diabetes mellitus, *n* (%)	18 (64.2)	6 (50.0)
**Hypertension**, ***n*** **(%)**	**20 (71.4)**	**4 (33.3)**
Advanced AAC	18 (64.3)	8 (66.7)
**VP (ng/mL)**	**180.4** **±** **54.8**	**145.1** **±** **59.7**
**Cardiovascular diseases**, ***n*** **(%)**	**22 (78.6)**	**3 (25.0)**
Systolic blood pressure (mmHg)	147.1 ± 22.6	130.8 ± 27.3
Diastolic blood pressure (mmHg)	73.5 ± 12.9	75.1 ± 15.6
Hemodialysis vintage (months)	79.5 ± 26.1	93.6 ± 28.5
Normalized protein catabolic rate (g/kg/day)	1.1 ± 0.3	1.1 ± 0.3
**Albumin (g/dL)**	**3.7** **±** **0.5**	**3.4** **±** **0.5**
Total cholesterol (mg/dL)	184.9 ± 31.9	179.9 ± 39.6
Triglyceride (mg/dL)	172.8 ± 116.4	186.9 ± 145.8
Blood urea nitrogen (mg/dL)	64.3 ± 17.0	60.2 ± 10.2
**Creatinine (mg/dL)**	**10.3** **±** **1.6**	**9.3** **±** **1.2**
Uric acid (mg/dL)	7.5 ± 1.2	7.20 ± 0.9
Potassium (mmol L^−1^)	4.3 ± 0.8	4.5 ± 0.8
Alanine aminotransferase (IU/L)	17.8 ± 7.1	15.0 ± 10.9
Alkaline phosphatase (IU/L)	90.1 ± 24.7	87.2 ± 22.7
Adjusted calcium (mg/dL)	8.9 ± 0.5	9.4 ± 0.9
Phosphate (mg/dL)	4.9 ± 1.6	4.6 ± 1.1
Calcium-phosphate product	43.5 ± 14.9	43.0 ± 10.1
Intact parathyroid hormone (pg/mL)	223.1 ± 246.6	298.4 ± 384.6
Hemoglobin (g/dL)	10.5 ± 1.2	10.8 ± 1.8
Platelet (k/μL)	211.6 ± 72.7	188.6 ± 59.8

*Continuous v ariables were expressed as mean ± SD. Categorical variables are expressed as n (%). Boldface indicates where the values differ significantly between survivors and non-survivors. AAC, aortic arch calcification; VP, vasopressin-neurophysin II-copeptin peptide*.

Table 4 summarizes the bivariate correlation coefficients between prognostic factors (VP and AAC) and bio-clinical parameters of interest in MHD patients at baseline. VP was significantly correlated with AAC (*r*, 0.28; *p* < 0.01), age (*r*, 0.25; *p* < 0.01), albumin (*r*, −0.20; *p* < 0.01), ALP (*r*, 0.23; *p* < 0.01), and previous CVD (*r*, 0.17; *p* < 0.05), respectively. AAC was in turn significantly correlated with age (*r*, 0.78; *p* < 0.01), ALP (*r*, 0.26; *p* < 0.01), and albumin (*r*, −0.22; *p* < 0.01). In the univariate Cox regression analysis of prognostic factors, higher VP levels, advanced AAC, previous CVD, DM, age, albumin, and ALP were significantly associated with all-cause mortality. Furthermore, higher VP levels, advanced AAC, previous CVD, DM, age, and ALP were significantly associated with CV mortality. The multivariable Cox regression model demonstrated higher VP, advanced AAC, previous CVD, DM, age, and albumin were still significantly associated with all-cause and CV mortality [aHR: 1.02 (95% confidence interval (CI): 1.01–1.02), 2.29 (95% CI: 1.14–4.59), 1.78 (95% CI: 1.10–3.53), 2.19 (95% CI: 1.09–4.37), 1.08 (95% CI: 1.01–1.17), 0.41 (95% CI: 0.21–0.82), and 1.00 (95% CI: 1.00–1.00), respectively]. However, we merely found that higher VP, advanced AAC, and age predicted CV mortality after multivariable adjustment [aHR: 1.03 (95% CI: 1.01–1.04), 2.40 (95% CI: 1.05–5.45), and 1.13 (95% CI: 1.02–1.26), respectively] (Tables 5, 6).

**Table 4:** The correlation analysis in VP, advanced AAC, and clinical parameters of interest.

	**VP**	**Advanced AAC**
VP	1.00	0.28^**^
AAC	0.28^**^	1.00
Diabetes mellitus	0.14	0.21^**^
Hypertension	0.07	0.10
Cardiovascular diseases	0.17^*^	0.16^*^
Age	0.25^**^	0.78^**^
Hemodialysis vintage	0.08	0.07
Normalized protein catabolic rate	− 0.09	− 0.15
Albumin	− 0.20^**^	− 0.22^**^
Creatinine	− 0.14	− 0.05
Total cholesterol	− 0.18	− 0.01
Triglyceride	− 0.08	− 0.03
Uric acid	0.03	− 0.13
Adjusted calcium	0.13	0.04
Phosphate	0.09	0.05
Alkaline phosphatase	0.23^**^	0.26^*^
Intact parathyroid hormone	0.11	0.13
Hemoglobin	0.02	− 0.01
Platelet	− 0.04	− 0.03

* *0.01 < p <0.05*,

** *p <0.01. AAC, aortic arch calcification; VP, vasopressin-neurophysin II-copeptin peptide*.

**Table 5:** Univariate and multivariable Cox regression analysis of prognostic factors for all-cause mortality.

	**Cox univariate**		**Cox multivariable**	
	**HR (95% CI)**	***p*-value**	**aHR (95% CI)**	***p-*value**
VP	1.014 (1.010–1.019)	*p* <0.01	1.016 (1.009–1.023)	*p* <0.05
ALP	1.020 (1.007–1.033)	*p* <0.01	1.006 (0.992–1.019)	*p* > 0.05
Age	1.091 (1.051–1.132)	*p* <0.01	1.084 (1.005–1.169)	*p* <0.05
Albumin	0.248 (0.123–0.503)	*p* <0.05	0.412 (0.207–0.822)	*p* <0.05
DM	2.250 (1.194–4.424)	*p* <0.05	2.186 (1.094–4.370)	*p* <0.05
CVD	2.066 (1.089–3.920)	*p* <0.05	1.781 (1.098–3.535)	*p* <0.05
Advanced AAC	3.303 (1.723–6.332)	*p* <0.01	2.286 (1.140–4.585)	*p* <0.05

*AAC, aortic arch calcification; ALP, alkaline phosphatase; CI, confidence interval; CVD, cardiovascular diseases; DM, diabetes mellitus; aHR, adjusted hazard ratio; VP, vasopressin-neurophysin II-copeptin peptide*.

**Table 6:** Univariate and multivariable Cox regression analysis of prognostic factors for CV mortality.

	**Cox univariate**		**Cox multivariable**	
	**HR (95% CI)**	***p*-value**	**aHR (95% CI)**	***p*-VALUE**
VP	1.017 (1.011–1.022)	*p* <0.01	1.025 (1.014–1.035)	*p* <0.01
ALP	1.016 (1.001–1.031)	*p* <0.05	1.002 (0.986–1.019)	*p* > 0.05
Age	1.103 (1.054–1.155)	*p* <0.01	1.131 (1.019–1.256)	*p* <0.05
Albumin	0.449 (0.190–1.061)	*p* > 0.05	0.748 (0.337–1.659)	*p* > 0.05
DM	2.664 (1.228–5.777)	*p* <0.05	2.010 (0.902–4.482)	*p* > 0.05
CVD	2.810 (1.828–6.127)	*p* <0.05	2.634 (0.959–6.715)	*p* > 0.05
Advanced AAC	3.150 (1.452–6.834)	*p* <0.01	2.396 (1.054–5.449)	*p* <0.05

*AAC, aortic arch calcification; ALP, alkaline phosphatase; CI, confidence interval; CVD, cardiovascular diseases; DM, diabetes mellitus; aHR, adjusted hazard ratio; VP, vasopressin-neurophysin II-copeptin peptide*.

### A Joint Evaluation of VP and AAC on All-Cause and CV Mortality Risks

**Figure 1:**
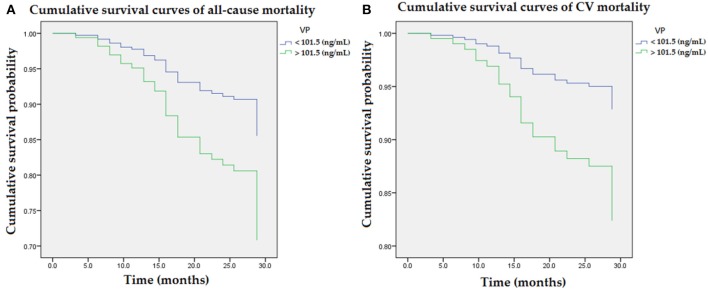
Cumulative survival curves of mortality risks with respect to plasma concentrations of VP after adjusting for advanced AAC, previous CVD, DM, age, albumin, and ALP during follow-up. **(A)** Higher concentration of VP (>101.5 ng/mL) was associated with an incremental risk of all-cause mortality. **(B)** The association between higher concentration of VP and CV mortality remained robust after multivariable adjustment. AAC, aortic arch calcification; ALP, alkaline phosphatase; CVD, cardiovascular diseases; DM, diabetes mellitus; VP, vasopressin-neurophysin II-copeptin peptide.

The interaction analysis between higher VP and advanced AAC was statistically significant for all-cause mortality and CV mortality (*p* < 0.05, respectively). Figure 1 illustrated cumulative survival curves of all-cause and CV mortality with respect to higher and lower categories of plasma VP after multivariable adjustment in the Cox regression model. Multivariable-adjusted results demonstrated higher VP concentrations were associated with incremental risks for all-cause and CV mortality [aHR: 2.2 (95% CI: 1.1–4.5) and 2.6 (95% CI: 1.1–4.6), respectively] in MHD patients.

**Figure 2:**
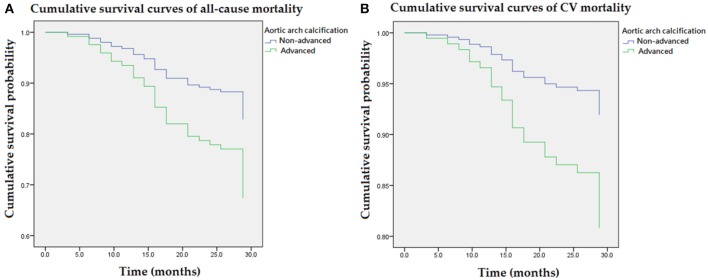
Cumulative survival curves of mortality risks with respect to advanced AAC after adjusting for higher VP levels, previous CVD, DM, age, albumin, and ALP during follow-up. **(A)** Advanced AAC was associated with an incremental risk of all-cause mortality. **(B)** The association between advanced AAC and CV mortality remained robust after multivariable adjustment. AAC, aortic arch calcification; ALP, alkaline phosphatase; CVD, cardiovascular diseases; DM, diabetes mellitus; VP, vasopressin-neurophysin II-copeptin peptide.

Figure 2 illustrated cumulative survival curves of all-cause and CV mortality with respect to advanced and non-advanced AAC after multivariable adjustment in the Cox regression model. Multivariable-adjusted results demonstrated advanced AAC was associated with incremental risks for all-cause and CV mortality [aHR: 2.1 (95% CI: 1.1–4.0) and 2.5 (95% CI: 1.0–4.3), respectively] in MHD patients.

**Figure 3:**
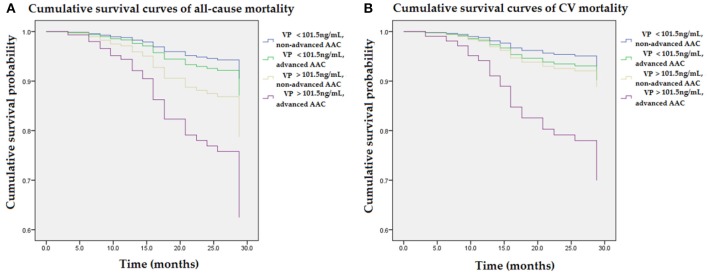
Cumulative survival curves of mortality risks with respect to four combined groups of VP and AAC after adjusting for higher VP levels, advanced AAC, previous CVD, DM, age, albumin, and ALP during follow-up. **(A)** The group with combined higher concentrations of VP (>101.5 ng/mL) and advanced AAC showed the highest risk for all-cause mortality as compared to the reference group (VP < 101.5 ng/mL and non-advanced AAC). **(B)** The group with combined higher concentrations of VP (>101.5 ng/mL) and advanced AAC showed the greatest CV mortality risk as compared to the reference group (VP < 101.5 ng/mL and non-advanced AAC). The interaction analysis between higher VP and advanced AAC remained statistically significant for all-cause mortality and CV mortality (*p* < 0.05, respectively). AAC, aortic arch calcification; ALP, alkaline phosphatase; CVD, cardiovascular diseases; DM, diabetes mellitus; VP, vasopressin-neurophysin II-copeptin peptide.

Figure 3 illustrated various mortality risks (aHR) compared to the reference group (VP < 101.5 ng/mL and non-advanced AAC) in the multivariable Cox regression model. Figure 3A illustrated mortality risks (aHR) for all-cause mortality in the non-advanced AAC and higher VP (>101.5 ng/mL) group, advanced AAC and lower VP (<101.5 ng/mL) group, advanced AAC and higher VP (>101.5 ng/mL) group were 1.4, 2.4, 4.7, respectively. Figure 3B illustrated mortality risks (aHR) for CV mortality in the non-advanced AAC and higher VP (>101.5 ng/mL) group, advanced AAC and lower VP (<101.5 ng/mL) group, advanced AAC and higher VP (>101.5 ng/mL) group were 1.4, 1.6, 4.9, respectively. Among four combined VP and AAC groups, patients with higher VP (>101.5 ng/mL) and advanced AAC had the greatest mortality risks for not only all-cause mortality but also CV mortality.

The data of receiver operating characteristics (ROC) curves were demonstrated in the Supplemental Figures 1–6. The ROC analysis for all-cause mortality showed that the area under curve (AUC) of higher VP was 0.725 (95% CI = 0.643–0.808, Supplemental Figure 1), and the AUC of advanced AAC was 0.745 (95% CI = 0.672–0.817, Supplemental Figure 2). Moreover, the combination of higher VP and advanced AAC yielded a higher AUC value at 0.769 (95% CI = 0.685–0.852, Supplemental Figure 3). On the other hand, the ROC analysis for CV mortality showed that the AUC of higher VP was 0.764 (95% CI = 0.694–0.834, Supplemental Figure 4), and the AUC of advanced AAC was 0.760 (95% CI = 0.691–0.830, Supplemental Figure 5). Furthermore, the combination of higher VP and advanced AAC yielded a higher AUC value at 0.801 (95% CI = 0.724–0.877, Supplemental Figure 6). In light of this, combined higher VP and advanced AAC as a predictor increases the sensitivity and specificity for all-cause and CV mortality.

## Discussion

The demand for earlier diagnosis, more precise prognostic evaluation and faster therapeutic decision-making process in diverse diseases has contributed to the ongoing investigations of novel biomarkers. The hope is that novel biomarkers will enable multidisciplinary specialist to identify the right level of care in clinical practice. In this cohort study of MHD patients, we show a brand new idea that a joint evaluation of VP and AAC provides more comprehensive warning effects and stronger predictive values for EOVC associated fatal events. In more depth, higher plasma concentrations of VP and advanced AAC interact to increase not only all-cause but also CV mortality. Several important findings in this work deserve further discussion.

**Figure 4:**
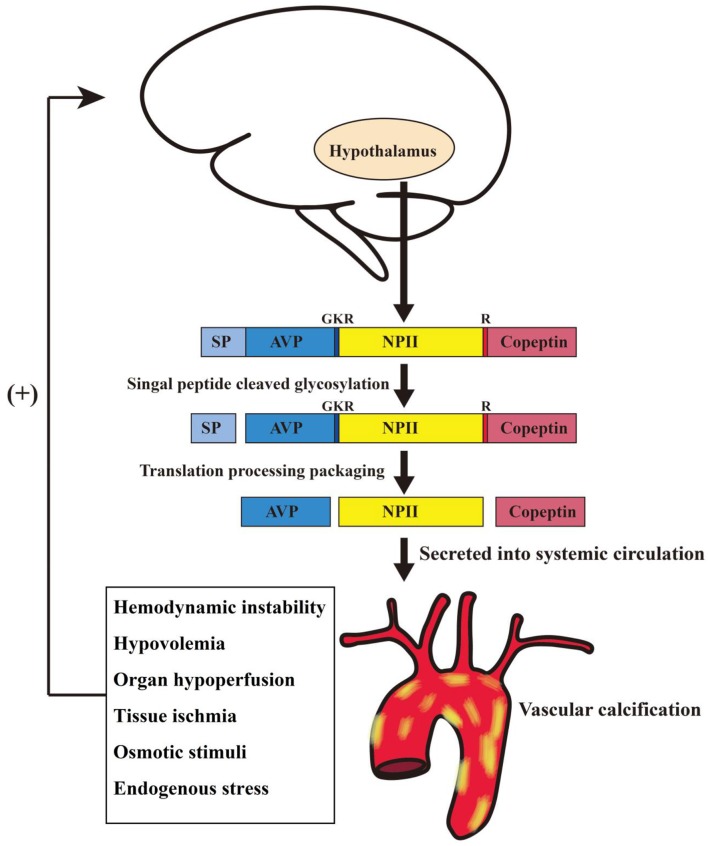
Schematic diagram illustrating possible synthesis and release mechanisms for vasopressin-neurophysin II-copeptin in hemodialysis patients with vascular calcification. AVP, arginine vasopressin; GKR, amino acid spacer; NP II, neurophysin II; R, monobasic cleavage site; SP, signal peptide.

VP is the key neurohormone derived from the magnocellular neurons of the hypothalamus. To a smaller extent, it is synthesized by other tissues including the sympathetic ganglia, adrenal glands, and the testis (27). VP is stored and secreted by granules within the posterior lobe of the pituitary, primarily as a response to hyperosmolarity, tissue hypoperfusion and low circulating effective volume (Figure 4). The synthesis of VP involves precursor peptides that are cleaved by a four-enzyme cascade into three major components: vasopressin, neurophysin II and copeptin. Since all the three cleavage products are generated in equal ratios (28), we examined the neurohormal effects of full-length peptide “vasopressin-neurophysin II-copeptin” in the current study.

Why is VP sufficiently specific as a biomarker of all-cause and CV mortality risk? The most powerful stimulus for VP secretion is the elevation in plasma osmolality, even 1% change is sufficient. VP is also released under diverse stressful stimuli involved in complex endocrine, autonomic, and behavioral responses. Adaptations to stress stimuli activate the HPA axis and the sympathetic nervous system, either acute or chronic (29, 30). In other words, circulating VP corresponds with human body's stress level. Even mild stress circumstance contributes to VP release. Because corticotropin releasing hormone and VP induce the generation of adrenocorticotropic hormone and cortisol in critical illness, the relationship between plasma osmolality and VP is lost. With the strong ability of vasoconstriction in peripheral vessels, VP is particularly produced in response to acute ischemic events, including hypovolemic shock and tissue hypoperfusion. Under critical conditions, plasma VP concentrations exceed the physiological range with exponential levels 100- or 1,000-fold times the normal range (28). Accordingly, VP acts as a versatile prognostic biomarker for various diseases, e.g., acute MI, heart failure, pulmonary diseases, acute dyspnea, sepsis, hemorrhagic shock, and ischemic stroke (5, 31, 32). Especially type 2 MI, known as secondary MI due to acute imbalance in oxygen supply and demand other than coronary artery occlusion, is more common in patients with renal failure (32). To the best of our knowledge, it is the first study to prove VP is intricately involved in EOVC related fatal ischemic events.

Why does VP interact with AAC to increase the risk of CV mortality? EOVC associated impaired vasodilation and luminal narrowing play pivotal roles in the pathophysiology of ischemic events and sudden cardiac death in MHD population (18), and AAC has been recognized as a potent indicator of EOVC (24). Zhang et al. conducted a meta-analysis to prove that AAC predicts CV and all-cause mortality in dialysis population (24). Since VP secretion is attributed to systemic hypoperfusion, a joint evaluation of VP and AAC is required for risk stratification. Given that both VP and AAC were intricately involved in EOVC related fatal events, we assumed that the mortality risk would be augmented among patients with higher levels of VP and AAC. In this study, the modification effect between AAC and VP on all-cause and CV mortality was examined using an interaction product term according to previous methods of moderation analysis (1, 19, 26, 33). As expected, the effects of VP on all-cause and CV mortality risks were modified by AAC in the multivariable model. Our data show that patients with higher plasma concentrations of VP (>101.5 ng/mL) and advanced AAC have the greatest risk of all-cause and CV death. Furthermore, the effects of higher VP on mortality are abated by non- advanced AAC, and vice versa. The interaction effect between VP and AAC on all-cause and CV mortality was statistically significant (*p* < 0.05 for the interaction term, respectively).

Our study has several limitations. First, our sample size was relatively small and our patients were only Asian patients, limiting the statistical power and generalization to other populations. Second, cross-sectional laboratory values might not reflect substantial intra-individual variability over time. Third, our blood samples were measured immediately due to the short half-life of vasopressin associated peptides, limiting the clinical practicality. Finally, prospective nonrandomized analysis is subject to residual confounders.

## Conclusions

Since risk stratification enables clinicians to identify the right level of care, searching for the novel biomarker is of prime importance for MHD patients with EOVC. Combined VP and AAC predict not only all-cause but also CV death in MHD patients, and a joint evaluation is more comprehensive than a single marker. In light of hypoperfusion and ischemic events in vital organs, VP and AAC could act as more robust dual marker for prognostic assessment.

## Data Availability Statement

The datasets generated for this study are available on request to the corresponding author.

## Ethics Statement

The studies involving human participants were reviewed and approved by The Research Ethics Review Committee of En Chu Kong Hospital (ECKIRB1070102). The written informed consent was obtained from the participants of this study.

## Author Contributions

Y-SC, J-FC, and T-MW were responsible for study concept and design, interpretation of data, writing of the manuscript, and drafting of the manuscript. J-CL and S-WC assisted with biochemical and digital analysis. T-HT and P-CC assisted with statistical analysis. L-LW, W-NL, and C-YH provided experimental supports of detection of biomarkers. W-CK and C-CW implemented the manuscript revision.

### Conflict of Interest

The authors declare that the research was conducted in the absence of any commercial or financial relationships that could be construed as a potential conflict of interest. The reviewer K-FH declared a past co-authorship with one of the authors C-YH to the handling Editor.

## References

[1] Ko WC Choy CS Lin WN Chang SW Liou JC Tung TH. Galectin-3 interacts with vascular cell adhesion molecule-1 to increase cardiovascular mortality in hemodialysis Patients. J Clin Med. (2018) 7:300. 10.3390/jcm7100300 30249969 PMC6213523

[2] Zhang Q Dong G Zhao X Wang M Li CS. Prognostic significance of hypothalamic-pituitary-adrenal axis hormones in early sepsis: a study performed in the emergency department. Intensive Care Med. (2014) 40:1499–508. 10.1007/s00134-014-3468-4 25223852

[3] Khan Sohail Q O'brien Russell J Struck J Quinn P Morgenthaler NG Squire Iain B Abstract 3391: C-terminal proVasopressin (Copeptin) as a novel and prognostic marker in acute myocardial infarction- the leicester acute myocardial infarction peptide (LAMP) study. Circulation. (2006) 114:II_721-II_721 10.1161/CIRCULATIONAHA.106.685503 17420344

[4] Voors AA Von Haehling S Anker SD Hillege HL Struck J Hartmann O. C-terminal provasopressin (copeptin) is a strong prognostic marker in patients with heart failure after an acute myocardial infarction: results from the OPTIMAAL study. Eur Heart J. (2009) 30:1187–94. 10.1093/eurheartj/ehp098 19346228

[5] Nickel CH Bingisser R Morgenthaler NG. The role of copeptin as a diagnostic and prognostic biomarker for risk stratification in the emergency department. BMC Med. (2012) 10:7–7. 10.1186/1741-7015-10-7 22264220 PMC3275505

[6] Liu LC Voors AA Valente MA Van Der Meer P. A novel approach to drug development in heart failure: towards personalized medicine. Can J Cardiol. (2014) 30:288–95. 10.1016/j.cjca.2013.12.005 24565253

[7] Mavani GP Devita MV Michelis MF. A review of the nonpressor and nonantidiuretic actions of the hormone vasopressin. Front Med. (2015) 2:19–19. 10.3389/fmed.2015.00019 25853137 PMC4371647

[8] Sriperumbuduri S Clark E Hiremath S. New insights into mechanisms of acute kidney injury in heart disease. Can J Cardiol. (2019) 35:1158–69. 10.1016/j.cjca.2019.06.032 31472814

[9] Jochberger S Morgenthaler NG Mayr VD Luckner G Wenzel V Ulmer H. Copeptin and arginine vasopressin concentrations in critically ill patients. J Clin Endocrinol Metab. (2006) 91:4381–6. 10.1210/jc.2005-2830 16940457

[10] Waters DD Arsenault BJ. Predicting prognosis in acute coronary syndromes: makeover time for TIMI and GRACE? Can J Cardiol. (2016) 32:1290–3. 10.1016/j.cjca.2016.02.053 27062237

[11] Morgenthaler NG Struck J Alonso C Bergmann A. Assay for the measurement of copeptin, a stable peptide derived from the precursor of vasopressin. Clin Chem. (2006) 52:112–9. 10.1373/clinchem.2005.060038 16269513

[12] Szinnai G Morgenthaler NG Berneis K Struck J Muller B Keller U. Changes in plasma copeptin, the c-terminal portion of arginine vasopressin during water deprivation and excess in healthy subjects. J Clin Endocrinol Metab. (2007) 92:3973–8. 10.1210/jc.2007-0232 17635944

[13] Morgenthaler NG Struck J Jochberger S Dunser MW. Copeptin: clinical use of a new biomarker. Trends Endocrinol Metab. (2008) 19:43–9. 10.1016/j.tem.2007.11.001 18291667

[14] Yalta K Yalta T Sivri N Yetkin E. Copeptin and cardiovascular disease: a review of a novel neurohormone. Int J Cardiol. (2013) 167:1750–9. 10.1016/j.ijcard.2012.12.039 23298558

[15] Ohtake T Kobayashi S Moriya H Negishi K Okamoto K Maesato K. High prevalence of occult coronary artery stenosis in patients with chronic kidney disease at the initiation of renal replacement therapy: an angiographic examination. J Am Soc Nephrol. (2005) 16:1141–8. 10.1681/ASN.2004090765 15743997

[16] Pun PH Smarz TR Honeycutt EF Shaw LK Al-Khatib SM Middleton JP. Chronic kidney disease is associated with increased risk of sudden cardiac death among patients with coronary artery disease. Kidney Int. (2009) 76:652–8. 10.1038/ki.2009.219 19536082 PMC2990680

[17] Wanner C Amann K Shoji T. The heart and vascular system in dialysis. Lancet. (2016) 388:276–84. 10.1016/S0140-6736(16)30508-6 27226133

[18] Makar MS Pun PH. Sudden cardiac death among hemodialysis patients. Am J Kidney Dis. (2017) 69:684–95. 10.1053/j.ajkd.2016.12.006 28223004 PMC5457912

[19] Chang J-F Feng Y-F Peng Y-S Hsu S-P Pai M-F Chen H-Y. Combined alkaline phosphatase and phosphorus levels as a predictor of mortality in maintenance hemodialysis patients. Medicine. (2014) 93:e106. 10.1097/MD.0000000000000106 25319440 PMC4616292

[20] Scialla JJ Kao WHL Crainiceanu C Sozio SM Oberai PC Shafi T. Biomarkers of vascular calcification and mortality in patients with ESRD. Clin J Am Soc Nephrol. (2014) 9:745–55. 10.2215/CJN.05450513 24458076 PMC3974354

[21] Iijima K Hashimoto H Hashimoto M Son BK Ota H Ogawa S. Aortic arch calcification detectable on chest X-ray is a strong independent predictor of cardiovascular events beyond traditional risk factors. Atherosclerosis. (2010) 210:137–44. 10.1016/j.atherosclerosis.2009.11.012 20006335

[22] Noordzij M Cranenburg EM Engelsman LF Hermans MM Boeschoten EW Brandenburg VM. Progression of aortic calcification is associated with disorders of mineral metabolism and mortality in chronic dialysis patients. Nephrol Dial Transplant. (2011) 26:1662–9. 10.1093/ndt/gfq582 20880929

[23] Abdelmalek JA Stark P Walther CP Ix JH Rifkin DE. Associations between coronary calcification on chest radiographs and mortality in hemodialysis patients. Am J Kidney Dis. (2012) 60:990–7. 10.1053/j.ajkd.2012.06.018 22883135 PMC3496035

[24] Zhang A Wang S Li H Yang J Wu H Aortic arch calcification and risk of cardiovascular or all-cause and mortality in dialysis patients: a meta-analysis. Sci Rep. (2016) 6:35375 10.1038/srep35375 27748417 PMC5066315

[25] Chang JF Hsu SP Pai MF Yang JY Chen HY Wu HY. High soluble vascular cell adhesion molecule-1 concentrations predict long-term mortality in hemodialysis patients. Int Urol Nephrol. (2013) 45:1693–701. 10.1007/s11255-013-0425-z 23563803

[26] Chang JF Yeh JC Chiu YL Liou JC Hsiung JR Tung TH. Periodontal pocket depth, hyperglycemia, and progression of chronic kidney disease: a population-based longitudinal study. Am J Med. (2017) 130:61–69.e61. 10.1016/j.amjmed.2016.08.024 27615146

[27] Francis LJ Perspectives on Vasopressin. London: World Scientific (2009).

[28] Bolignano D Cabassi A Fiaccadori E Ghigo E Pasquali R Peracino A. Copeptin (CTproAVP), a new tool for understanding the role of vasopressin in pathophysiology. Clin Chem Lab Med. (2014) 52:1447–56. 10.1515/cclm-2014-0379 24940718

[29] Dedovic K Duchesne A Andrews J Engert V Pruessner JC. The brain and the stress axis: the neural correlates of cortisol regulation in response to stress. Neuroimage. (2009) 47:864–71. 10.1016/j.neuroimage.2009.05.074 19500680

[30] Kvetnansky R Sabban EL Palkovits M. Catecholaminergic systems in stress: structural and molecular genetic approaches. Physiol Rev. (2009) 89:535–606. 10.1152/physrev.00042.2006 19342614

[31] Dobsa L Edozien KC. Copeptin and its potential role in diagnosis and prognosis of various diseases. Biochem Med. (2013) 23:172–90. 10.11613/BM.2013.021 23894863 PMC3900057

[32] Thygesen K Alpert JS Jaffe AS Chaitman BR Bax JJ Morrow DA Fourth universal definition of myocardial infarction 2018. Eur Heart J. (2019) 40:237–69. 10.1093/eurheartj/ehy462 30165617

[33] Yeh J-C Wu C-C Choy C-S Chang S-W Liou J-C Chen K-S. Non-hepatic alkaline phosphatase, hs-CRP and progression of vertebral fracture in patients with rheumatoid arthritis: a population-based longitudinal study. J Clin Med. (2018) 7:439. 10.3390/jcm7110439 30428612 PMC6262279

