# Supplementary figures and images for: A Joint Evaluation of Neurohormone Vasopressin-Neurophysin II-Copeptin and Aortic Arch Calcification on Mortality Risks in Hemodialysis Patients
Source: Front Med (Lausanne). 2020 Mar 31;7:102. doi: 10.3389/fmed.2020.00102 (PMC7136408; doi:10.3389/fmed.2020.00102)

Supplemental Figure 1. ROC analysis for all-cause mortality using higher VP as a predictor.

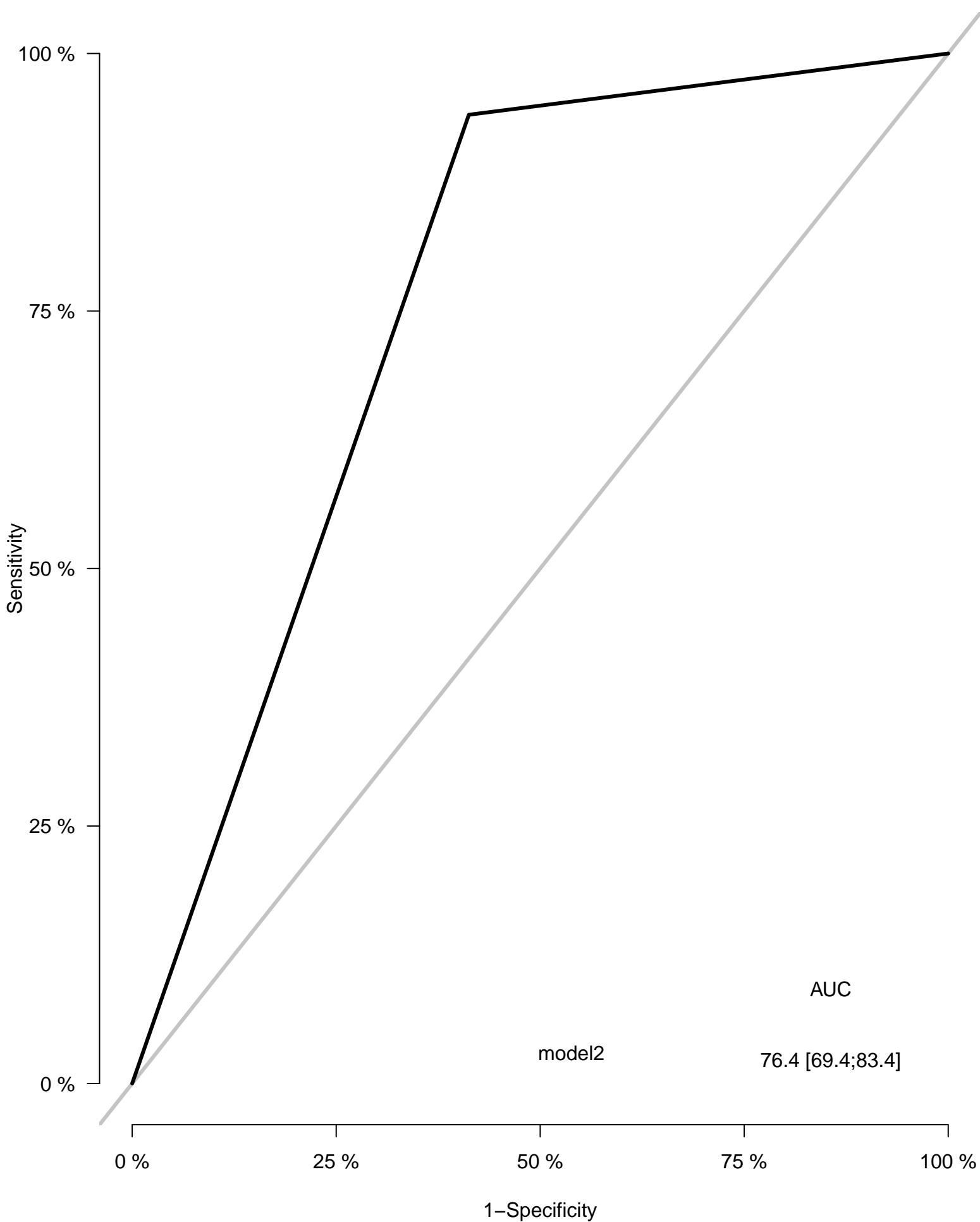

Supplement: Supplementary file 1 [file Image_1.pdf]

Supplemental Figure 2. ROC analysis for all-cause mortality using advanced AAC as a predictor.

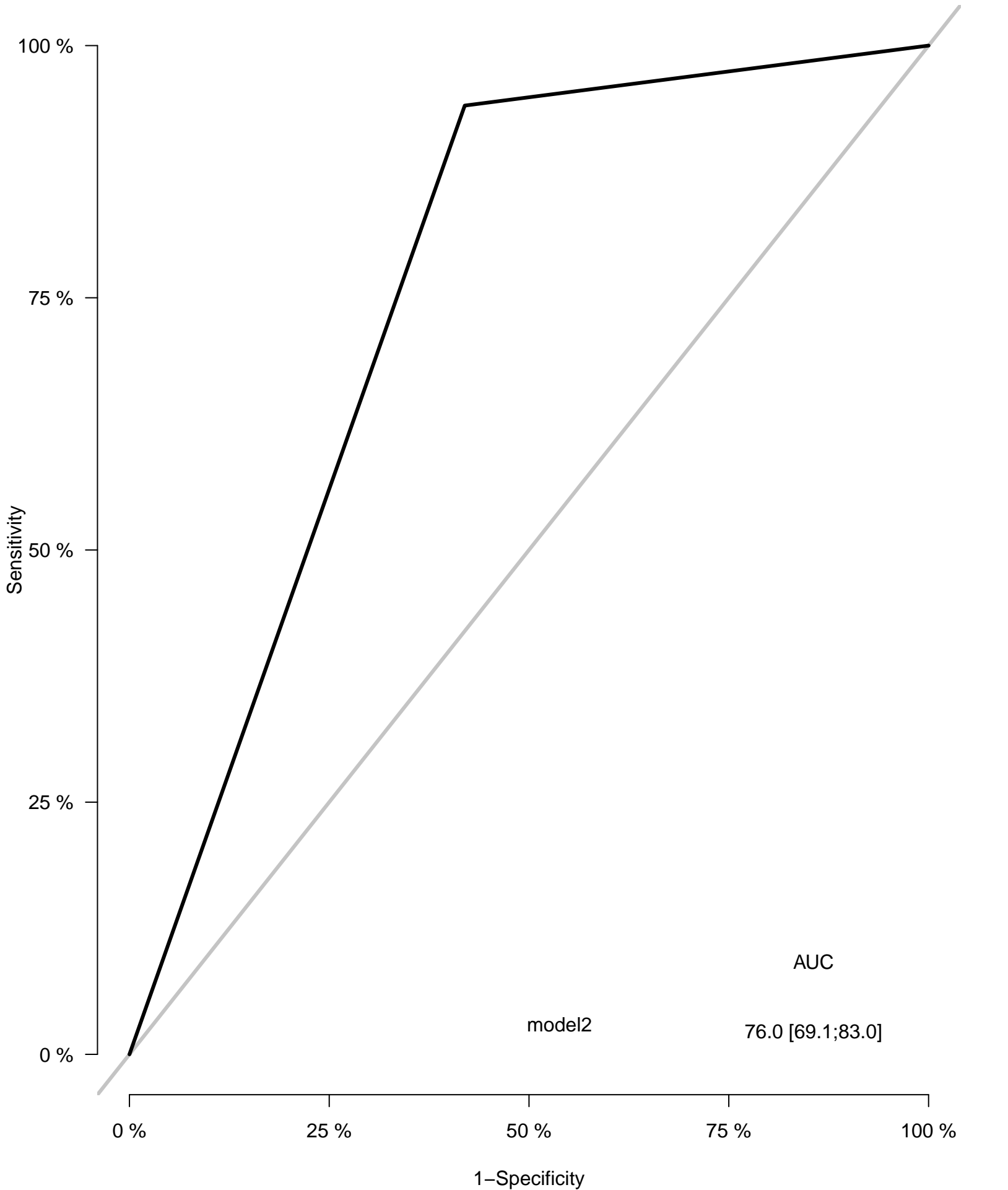

Supplement: Supplementary file 2 [file Image_2.pdf]

Supplemental Figure 4. ROC analysis for CV mortality using higher VP as a predictor.

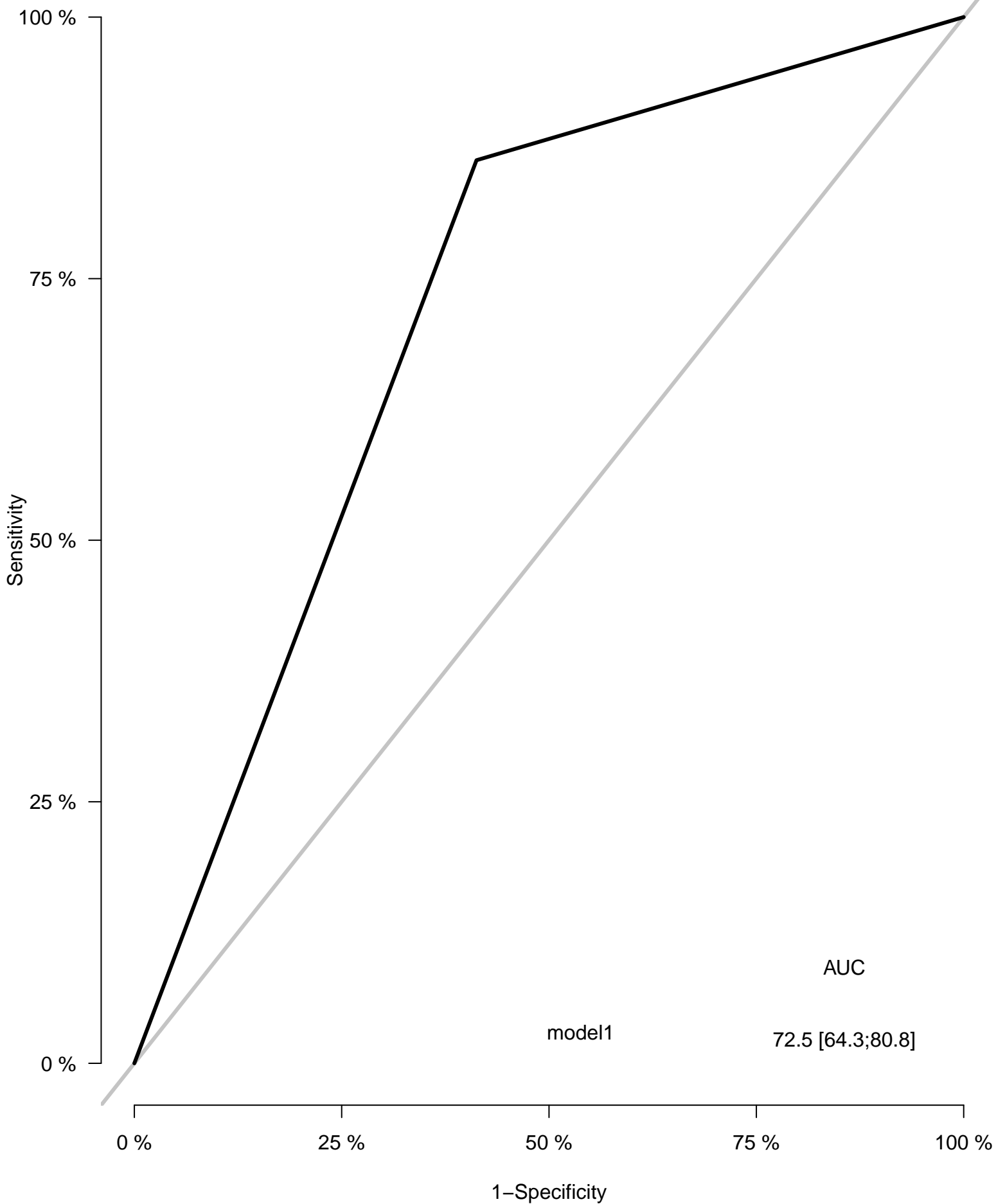

Supplement: Supplementary file 4 [file Image_4.pdf]

Supplemental Figure 5. ROC analysis for CV mortality using advanced AAC

as a predictor.

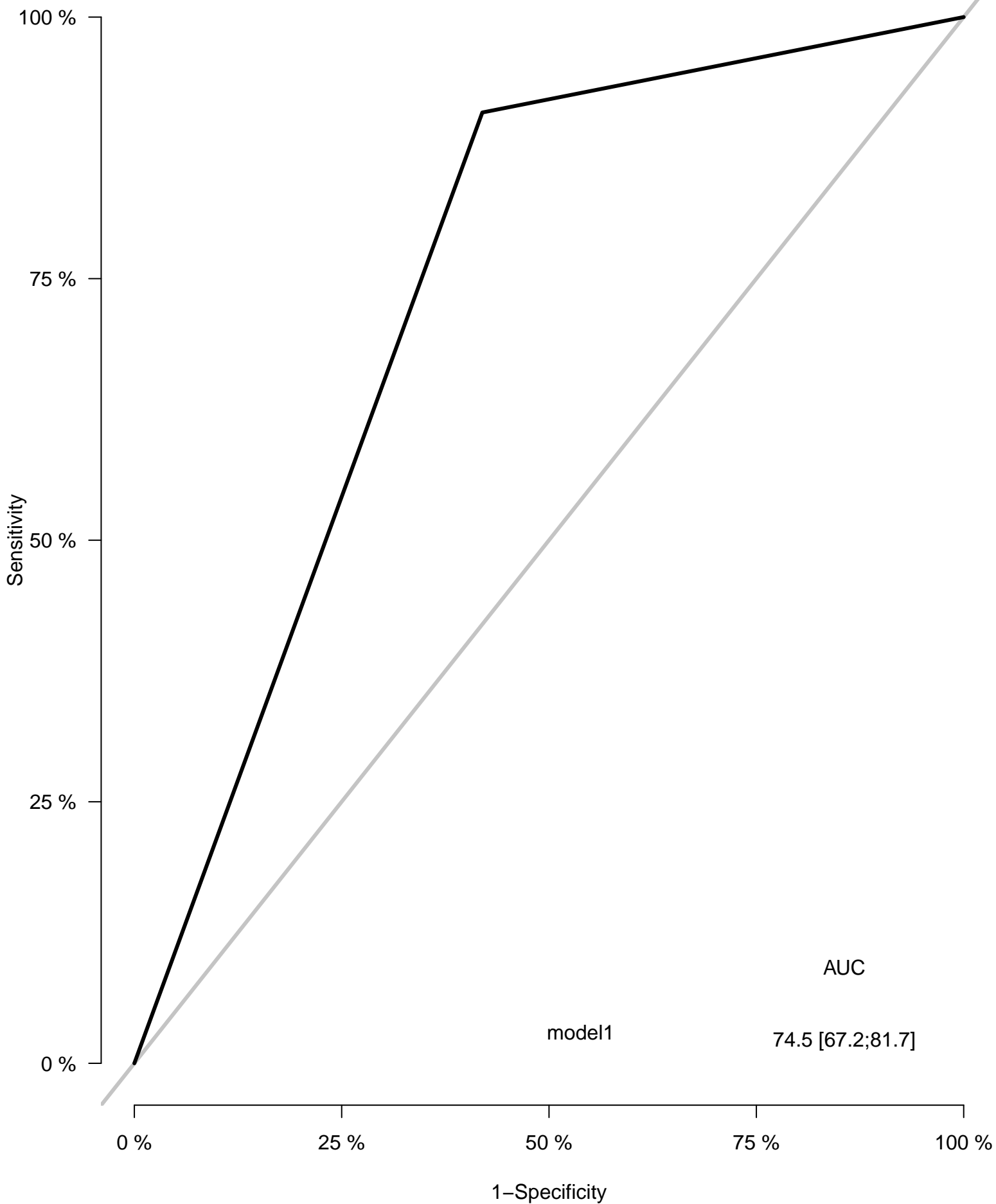

Supplement: Supplementary file 5 [file Image_5.pdf]
