# Supplementary material for: A Joint Evaluation of Neurohormone Vasopressin-Neurophysin II-Copeptin and Aortic Arch Calcification on Mortality Risks in Hemodialysis Patients
Source: Front Med (Lausanne). 2020 Mar 31;7:102. doi: 10.3389/fmed.2020.00102 (PMC7136408; doi:10.3389/fmed.2020.00102)

Supplemental Figure 3. ROC analysis for all-cause mortality using the combination of higher VP and advanced AAC as a predictor.

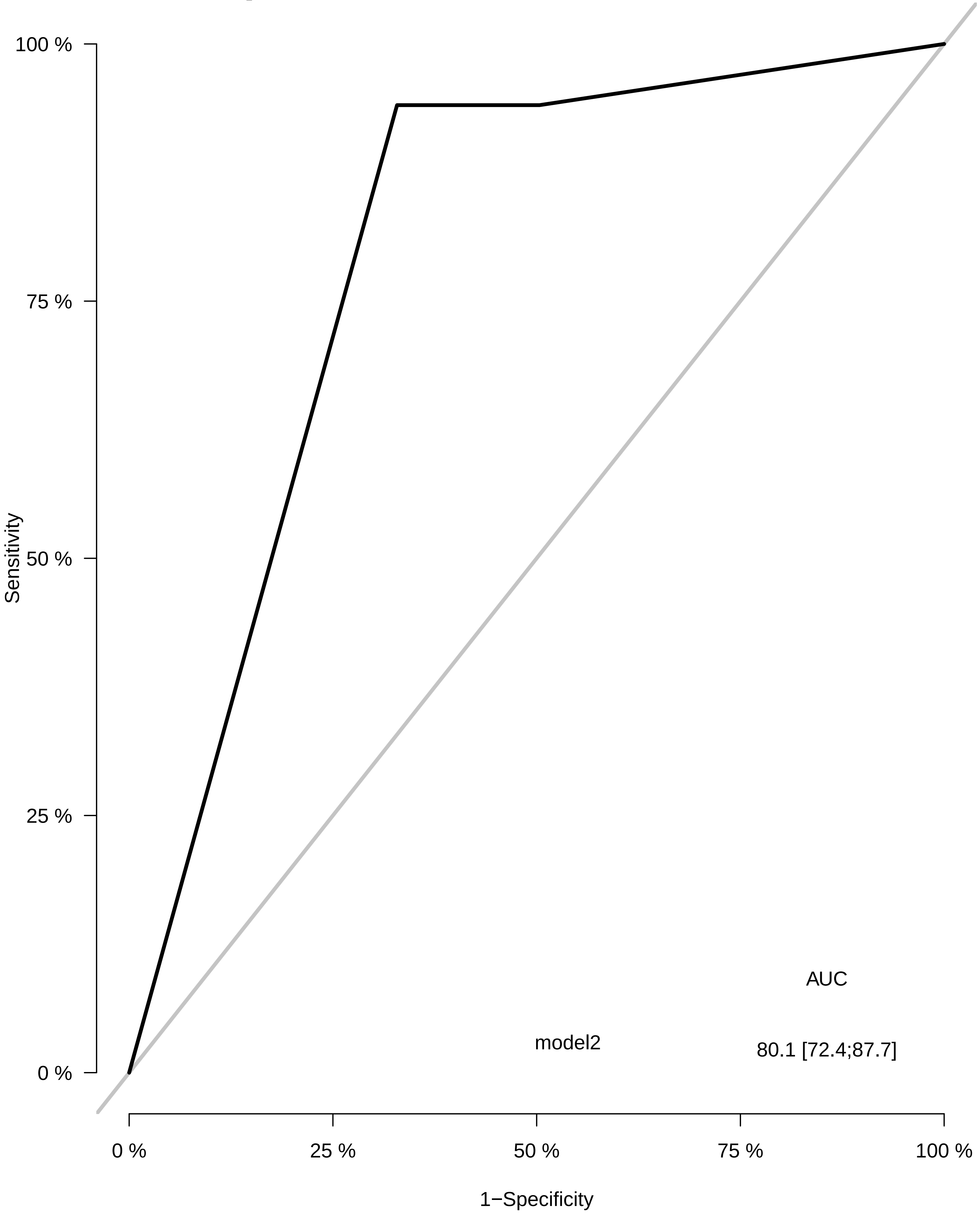

Supplement: Supplementary file 3 [file Image_3.pdf]
